# Chronic leucine supplementation improves lipid metabolism in C57BL/6J mice fed with a high-fat/cholesterol diet

**DOI:** 10.3402/fnr.v60.31304

**Published:** 2016-09-09

**Authors:** Jun Jiao, Shu-Fen Han, Wei Zhang, Jia-Ying Xu, Xing Tong, Xue-Bin Yin, Lin-Xi Yuan, Li-Qiang Qin

**Affiliations:** 1 Department of Nutrition and Food Hygiene, School of Public Health, Soochow University, Suzhou, China; 2 School of Radiation Medicine and Protection, Soochow University, Suzhou, China; 3 Collaborative Innovation Center of Radiation Medicine of Jiangsu Higher Education Institutions, Soochow University, Suzhou, China; 4 Jiangsu Bio-Engineering Research Centre of Selenium, Suzhou, China; 5 Jiangsu Key Laboratory of Preventive and Translational Medicine for Geriatric Disease, Soochow University, Suzhou, China

**Keywords:** leucine, lipid metabolism, brown adipose tissue, white adipose tissue, WAT browning

## Abstract

**Background:**

Leucine supplementation has been reported to improve lipid metabolism. However, lipid metabolism in adipose tissues and liver has not been extensively studied for leucine supplementation in mice fed with a high-fat/cholesterol diet (HFCD).

**Design:**

C57BL/6J mice were fed a chow diet, HFCD, HFCD supplemented with 1.5% leucine (HFCD+1.5% Leu group) or 3% leucine (HFCD+3% Leu group) for 24 weeks. The body weight, peritoneal adipose weight, total cholesterol (TC), triglyceride in serum and liver, and serum adipokines were analyzed. In addition, expression levels of proteins associated with hepatic lipogenesis, adipocyte lipolysis, and white adipose tissue (WAT) browning were determined.

**Results:**

Mice in the HFCD group developed obesity and deteriorated lipid metabolism. Compared with HFCD, leucine supplementation lowered weight gain and TC levels in circulation and the liver without changing energy intake. The decrease in body fat was supported by histological examination in the WAT and liver. Furthermore, serum levels of proinflammatory adipokines, such as leptin, IL-6, and tumor necrosis factor-alpha, were significantly decreased by supplemented leucine. At the protein level, leucine potently decreased the hepatic lipogenic enzymes (fatty acid synthase and acetyl-coenzyme A carboxylase) and corresponding upstream proteins. In epididymal WAT, the reduced expression levels of two major lipases by HFCD, namely phosphorylated hormone-sensitive lipase and adipose triglyceride lipase, were reversed when leucine was supplemented. Uncoupling protein 1, β3 adrenergic receptors, peroxisome proliferator-activated receptor g coactivator-1α, and fibroblast growth factor 21 were involved in the thermogenic program and WAT browning. Leucine additionally upregulated their protein expression in both WAT and interscapular brown adipose tissue.

**Conclusion:**

This study demonstrated that chronic leucine supplementation reduced the body weight and improved the lipid profile of mice fed with a HFCD. This beneficial effect was ascribed to hepatic lipogenesis, adipocyte lipolysis, and WAT browning.

The prevalence of obesity is increasing at an alarming rate in many parts of the world. About 2 billion people are overweight, and one-third of them are obese (1). Obesity is a major health problem because it increases the risk of several clinical conditions, including diabetes and cardiovascular diseases (2, 3). From a public health perspective, the current obesity epidemic must be reduced. Besides regular physical activity, choosing healthy food is the main way to prevent obesity (4). In this case, manipulating the dietary composition of macronutrients merits careful investigation because macronutrients not only provide calories, but they also work as signaling molecules that can affect cellular metabolic processes (5–7). Leucine, which is an essential branched chain amino acid (BCAA), is one such example (8, 9). Our multi-ethnic population-based international study also demonstrated that the intake of BCAA including leucine is inversely associated with the prevalence of overweight status and obesity (10).

In 2006, Cota found that the central administration of leucine decreases food intake and body weight in rats by stimulating the intracellular energy-sensing mammalian target of rapamycin (mTOR) pathway (7). Consequently, Zhang found that increasing leucine intake for 14 weeks results in reduced weight and adiposity in high-fat diet (HFD)-fed mice (11). Since then, several studies have observed the effects of leucine supplementation on HFD-induced obesity and metabolic profile through multiple mechanisms, but the results were inconsistent (12–19). Obesity results from the expansion of adipose tissue, which is categorically divided into white adipose tissue (WAT) and brown adipose tissue (BAT). WAT is characterized by lipid storage as triglyceride and endocrine functions. In this case, the promotion of lipolysis in WAT is beneficial for the prevention and treatment of obesity. Meanwhile, the main BAT function is energy dissipation, which is largely in the form of heat (20). Certain white depots have been found to develop brownish characteristics (21). Thus, inducible brown-like adipocytes from WAT (WAT browning) may be a promising strategy to combat obesity.

Despite the intricate mechanisms dictating obesity, lipid metabolism in adipose tissues itself and liver, other major site for lipid metabolism has not been extensively studied for the beneficial effect of leucine supplementation on obesity prevention. Therefore, we investigated whether or not chronic leucine supplementation with a high-fat/cholesterol diet (HFCD) in rats has a direct action on target tissues, focusing on the expression of proteins related to hepatic lipogenesis, adipocyte lipolysis, and WAT browning.

## Materials and methods

### Experimental animals and diets

Seven-week-old male C57BL/6J mice were purchased from SLAC Laboratory Animal Company (Shanghai, China). The mice were housed in standard cages (four to five mice per cage) with a constant temperature of 22°C±2°C, 60% relative humidity, and an artificial 12-h light–darkness cycle. All of the animal studies were pre-approved by the Soochow University Animal Welfare Committee. After 1 week of acclimatization to the laboratory conditions, the animals were weighed and randomly assigned to one of four dietary groups (14 mice per group), namely those fed with chow diet (CD group), HFCD group, HFCD supplemented with 1.5% leucine (HFCD+1.5% Leu group) or 3.0% leucine (HFCD+3% Leu group). The CD contained 3.90 kcal/g with 20.8, 67.7, and 11.5% calories from protein, carbohydrates, and fat, respectively. The HFCD obtained from Research Diets Inc. (New Brunswick, NJ, USA, D12451+1% cholesterol) was known to induce obesity and dyslipidemia. It contained 4.77 kcal/g with 19.6, 34.4, and 46% calories from protein, carbohydrates, and fat, respectively. l-leucine (Luzhou Amino Acid Co., Ltd, Shandong, China) was directly mixed with the HFCD by percentage weight in supplemental diets. The animals had free access to food and water. The experiment lasted for 24 weeks. Body weight and food intakes were routinely recorded on a weekly basis. The average calorie intake was calculated by food intake.

### Tissue sample and blood management

Blood samples were obtained from the angular vein after 12 h of fasting every 8 weeks including the start and end of the experiment (weeks 0, 8, 16, and 24). Serum was separated and stored at −80°C in a freezer until analysis. At the 24th week of the experiment, the animals were sacrificed under mild ether anesthesia. Peritoneal adipose tissue (excepting mesenteric) was removed and weighted. The liver, epididymal WAT, and interscapular BAT were immediately collected, frozen in liquid nitrogen, and stored at −80°C in a freezer for further analyses.

### Histological examination

Histological examination of livers and WAT samples was carried out using the HE staining method. The samples were fixed in 10% neutral buffered formalin for 6 h and dehydrated as standard before embedding in paraffin wax. Sections were cut into 4-µm slices and mounted on polylysine-handled glass slides, and HE staining was performed.

### Biochemical analysis in serum and liver

Serum levels of total cholesterol (TC) and triglyceride (TG) at each time point were determined by enzymatic methods using commercial kits (Applygen Technologies Inc., Beijing, China). The serum sample at the end of the experiment was also used to determine glucose (Applygen Technologies Inc., Beijing, China), insulin (Mercodia AB, Sweden), and adipocytokines including interleukin-6 (IL-6), leptin, tumor necrosis factor-alpha (TNF-α), and adiponectin with Luminex^®^ xMAP Technology (Merck Millipore Bioscience, USA). The insulin resistance was estimated by homeostasis model assessment-insulin resistance (HOMA-IR) using the following formula: HOMA-IR=fasting glucose (mmol/L)×insulin (pmol/L)/22.5. Parts of the liver tissue were homogenated. The hepatic levels of TG and TC were analyzed with commercialized kits (Applygen Technologies Inc., Beijing, China) and normalized to protein levels.

### Western blot analysis

The hepatic tissue and epididymal and interscapular adipose tissue samples were homogenized in lysis buffer (Beyotime Institute of Biotechnology, Nantong, China). Protein concentration was determined according to the bicinchoninic acid assay (BCA) protein assay (Beyotime Institute of Biotechnology, Nantong, China). Equal amounts of protein (30–50 µg) were loaded to a 12% sodium dodecylsulfate (SDS)-polyacrylamide gel and then transferred to a polyvinylidene fluoride (PVDF) membrane (EMD Millipore Corporation, CA, USA) through electrophoretic transfer. Subsequently, non-specific binding sites were blocked with 5% fat-free dry milk in phosphate-buffered saline containing 0.1% Tween-20 for 1 h at room temperature. The blots were incubated overnight at 4°C with primary antibodies according to the recommendation of the manufacturer. The antibodies for hepatic tissues included fatty acid synthase (FAS, Cell Signaling Technology, CST3180, 1:1000), acetyl-coenzyme A carboxylase (ACC, CST3676, 1:1000), sterol regulatory element binding protein-1 (SREBP-1, Abcam ab28481, 1:500), and liver X receptorα (LXRα, Abcam ab176323, 1:1000). Hormone-sensitive lipase (HSL, Abcam ab45422, 1:1000), p-HSL (Ser563, CST4139, 1:1000), p-HSL (Ser660, CST4139, 1:1000), adipose triglyceride lipase (ATGL, CST2138, 1:1000), and cAMP-dependent protein kinase (PKA, YT 374, 1:1000) were used in epididymal adipose tissues. Meanwhile, uncoupling protein 1 (UCP1, Abcamab10983), β3 adrenergic receptors (β3AR, YT 0363, 1:1000), peroxisome proliferator-activated receptor g coactivator-1α+β (PGC-1α+β, Abcam ab72230, 1:1000), and fibroblast growth factor 21 (FGF-21, Abcam ab171941, 1:1000) were used in epididymal and interscapular adipose tissues. After washing for three times, the antigen–antibody complexes were visualized for 1–1.5 h at room temperature with Peroxidase AffiniPure goat anti-mouse or anti-rabbit IgG antibody (Jackson ImmunoResarch Laboratories, Inc., Canada). Antibody reactivity was detected by chemiluminescence using enhanced chemiluminescent (ECL) Detection Systems (EMD Millipore Corporation, CA, USA). Blots were performed at least three times to confirm the reproducibility of the results. The intensity of the bands was normalized using each corresponding β-actin density as an internal control.

### Statistical analysis

Data are expressed as the mean±standard deviation. All the analyses were performed using SPSS version 16.0 statistical analysis package (SPSS Inc., Chicago, IL, USA). The significance of differences among the four dietary groups was assessed by one-way ANOVA, followed by Fisher's least significant difference (LSD) *post hoc* test. Differences were considered significant when *p*<0.05.

## Results

**Fig. 1:**
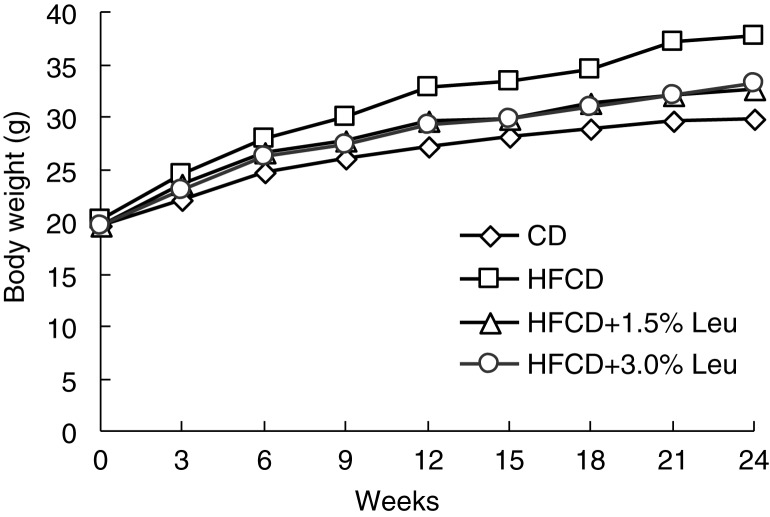
Chronic leucine supplementation decreases the body weight in HFCD-fed mice from week 9. (*n*=14 for each group). CD, chow diet; HFCD, high-fat/cholesterol diet; Leu, leucine.

Mice in the HFCD group grew fast during the entire study course and exhibited increased body weight of 71.89% higher than those in the CD group at the end of the experiment. The mice in the two leucine groups had comparable body weights, but they were heavier than those in the CD group and significantly lower than those in the HFCD group from week 9 (Fig. 1). At the end of the experiment, the weight gain of the mice was 9.21±1.46, 17.55±2.60, 12.94±2.22, and 13.78±2.50 g in the CD group, HFCD group, HFCD+1.5% Leu group, and HFCD+3.0% Leu group, respectively. Weight gain was significantly lower in the supplemented leucine groups than in the HFCD group. The mice fed with HFCD with and without leucine ingested less food than the mice in the CD group. However, when expressed in kcal/day, energy intake by the four groups was comparable. HFCD feeding significantly increased peritoneal adipose tissue and liver weights. Supplemental leucine significantly decreased their weights compared with the HFCD group; however, there were no significant differences in the ratios of fat and liver weights to body weight between HFCD with and without leucine (Table 1).

**Table 1:** Effects of leucine supplementation on food and energy intake, body and organ weight in C57BL/6J mice

	Food intake (g/day)	Energy (kcal/day)	Weight gain (g)	Fat weight^a^ (g)	Fat weight/BW (%)	Liver weight (g)	Liver weight/BW (%)
CD	3.14±0.23	12.26±0.89	9.21±1.46	0.57±0.22	1.89±0.70	1.16±0.10	3.89±0.26
HFCD	2.57±0.22**	12.25±1.08	17.55±2.60**	2.29±0.62**	6.02±1.26**	1.44±0.15*	3.80±0.17
HFCD+1.5% leucine (Leu)	2.52±0.30**	11.98±1.41	12.94±2.22*,#	1.70±0.84**,#	5.24±1.79**	1.29±0.11*,#	3.89±0.39
HFCD+3% Leu	2.59±0.30**	12.31±1.44	13.78±2.50*,#	1.71±0.85**,#	4.74±1.72**	1.24±0.09#	3.53±0.50

Values are mean±SD (*n*=14).

* *p*<0.05 versus CD group

** *p*<0.01 versus CD group

# *p*<0.05 versus HFCD group.

a Peritoneal adipose tissue (excepting mesenteric).

CD, chow diet; HFCD, high-fat/cholesterol diet; BW, body weight.

**Fig. 2:**
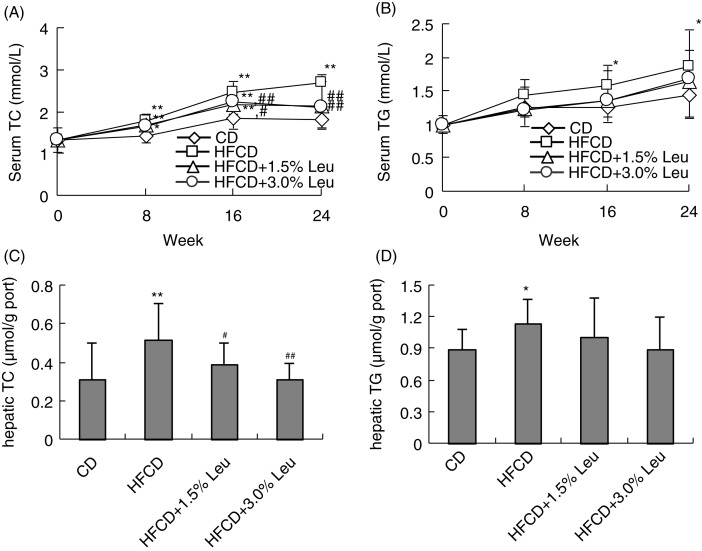
Chronic leucine supplementation decreases serum and hepatic TC levels in mice on HFCD. Values are means for 14 rats with SD represented by vertical bars. *p<0.05 versus CD group, **p<0.01 versus CD group, ^#^ *p*<0.05 versus HFCD group, ^##^ *p*<0.01 versus HFCD group. CD, chow diet; HFCD, high-fat/cholesterol diet; Leu, leucine; TC, total cholesterol; TG, triglyceride.

As expected, serum TC and TG were significantly higher in the HFCD group than in the CD group. Serum TC was significantly lower in the two leucine groups than in the HFCD group at weeks 16 and 24. At the end of the experiment (week 24), the serum TC levels were 1.71±0.20, 2.69±0.45, 2.15±0.54, and 2.12±0.60 mmol/L. Leucine supplementation mitigated serum TG compared with the HFCD group, but no significant difference was observed. For hepatic lipids, TC levels in the liver homogenate were significantly increased by HFCD (0.31±0.19 µmol/g vs. 0.52±0.19 µmol/g) and significantly decreased to a level similar to the CD group by supplemental 1.5% leucine (0.39±0.11 µmol/g) and 3.0% leucine (0.31±0.09 µmol/g). Meanwhile, hepatic TG was slightly increased by HFCD, and its decrease through leucine supplementation was not significant. The levels of TC and TG in circulation and in the liver were comparable in both leucine supplementation groups (Fig. 2).

HFCD resulted in a significant increase in fasting glucose and insulin. Although fasting glucose levels were comparable regardless of whether leucine was supplemented, leucine supplementation caused significantly lower fasting plasma insulin, leading to a decreased HOMA index in the leucine-supplemented group compared with the HFCD group. We also determined several notable adipokines implicated in obesity and obesity-related metabolic disorders, which included leptin, IL-6, TNF-α, and adiponectin. HFCD significantly increased serum levels of leptin, IL-6, and TNF-α and decreased adiponectin. Compared with the HFCD group, leptin level was significantly decreased in the HFC+1.5% Leu and HFC+3.0% Leu groups, and IL-6 and TNF-α levels were significantly decreased in the HFC+3.0% Leu group. However, no difference in adiponectin level was observed between HFCD group and HFCD groups supplemented with leucine (Table 2).

**Table 2:** Effects of leucine supplementation on glucose metabolism and serum adipokines in C57BL/6J mice

	Insulin (µg/L)	Glucose (mmol/L)	HOMA-IR	Leptin (ng/mL)	Adiponectin (ng/mL)	IL-6 (pg/mL)	TNF-α (pg/mL)
CD	0.55±0.09	6.66±1.18	3.43±0.80	0.73±0.42	16.71±1.66	3.64±1.80	2.67±0.86
HFCD	0.75±0.22*	8.21±2.06*	6.41±2.00**	7.14±3.44**	13.88±1.51**	7.93±3.16**	3.86±1.45**
HFCD+1.5% leucine (Leu)	0.55±0.11##	7.98±1.91	3.65±1.32##	4.21±2.37**,##	15.34±2.03	6.93±1.52**	3.22±0.82
HFCD+3% Leu	0.61±0.16,#	7.94±1.22	4.72±1.76*,##	4.24±2.22**,##	14.89±2.41	6.15±1.87**,#	2.85±0.76,#

Values are mean±SD (*n*=14).

* *p*<0.05 versus CD group

** *p*<0.01 versus CD group

# *p*<0.05 versus HFCD group

## *p*<0.05 versus HFCD group.

CD, chow diet; HFCD, high-fat/cholesterol diet; HOMA-IR, homeostasis model assessment-insulin resistance; TNF-α, tumor necrosis factor-alpha.

**Fig. 3:**
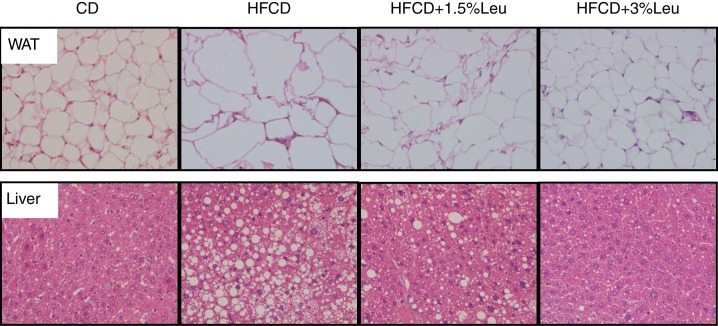
Histological examination (400×) showed that adipocyte size was markedly reduced, and hepatic lipid accumulation was alleviated after leucine supplementation. CD, chow diet; HFCD, high-fat/cholesterol diet; Leu, leucine; WAT, white adipose tissue.

Histological examination showed that HFCD enlarged the adipocyte size of WAT and caused the hepatic lipids to accumulate. However, adipocyte size was markedly reduced, and hepatic lipid accumulation was alleviated after leucine supplementation (Fig. 3).

**Fig. 4:**
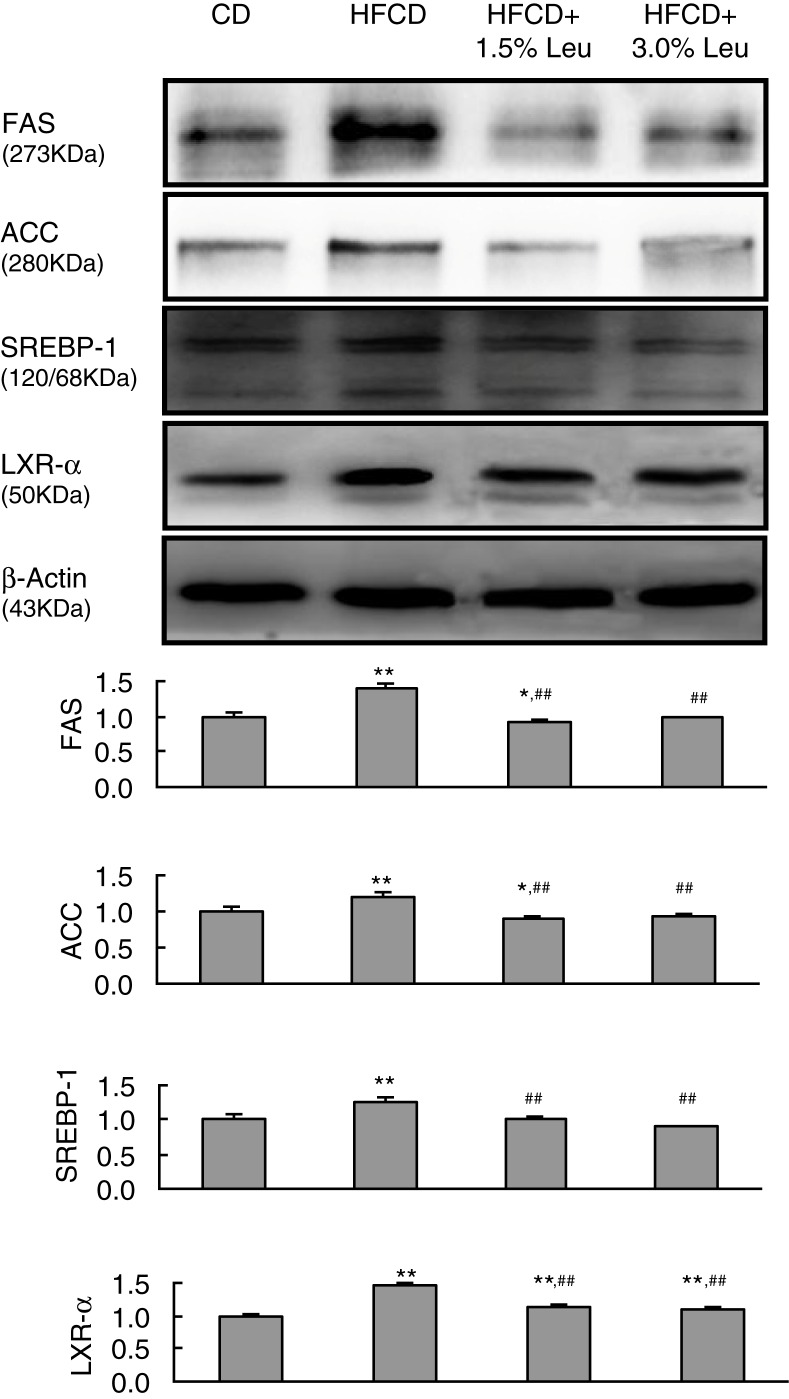
Chronic leucine supplementation improves lipid metabolism by decreased hepatic lipogenesis in mice fed with HFCD. Values are means for three rats with SD represented by vertical bars. *p<0.05 versus CD group, **p<0.01 versus CD group, ^##^ *p*<0.01 versus HFCD group. CD, chow diet; HFCD, high-fat/cholesterol diet; Leu, leucine; FAS, fatty acid synthase; ACC, acetyl-coenzyme A carboxylase; SREBP-1, sterol regulatory element binding protein-1; LXRα, liver X receptor α.

To provide molecular evidence for the role of leucine in hepatic lipid metabolism, protein expression of lipogenic enzymes, including FAS and ACC, was measured. As a result, HFCD increased FAS and ACC expression, and the overexpression was retarded by additional leucine supplementation. On the contrary, ACC and FAS were regulated by SREBP-1, which is strongly activated by LXRα. Similarly, the overexpression of LXRa and SREBP-1 by HFCD was reverted by leucine supplementation. The protein expression was even lower in the HFCD+3.0 Leu group than in the CD group (Fig. 4).

**Fig. 5:**
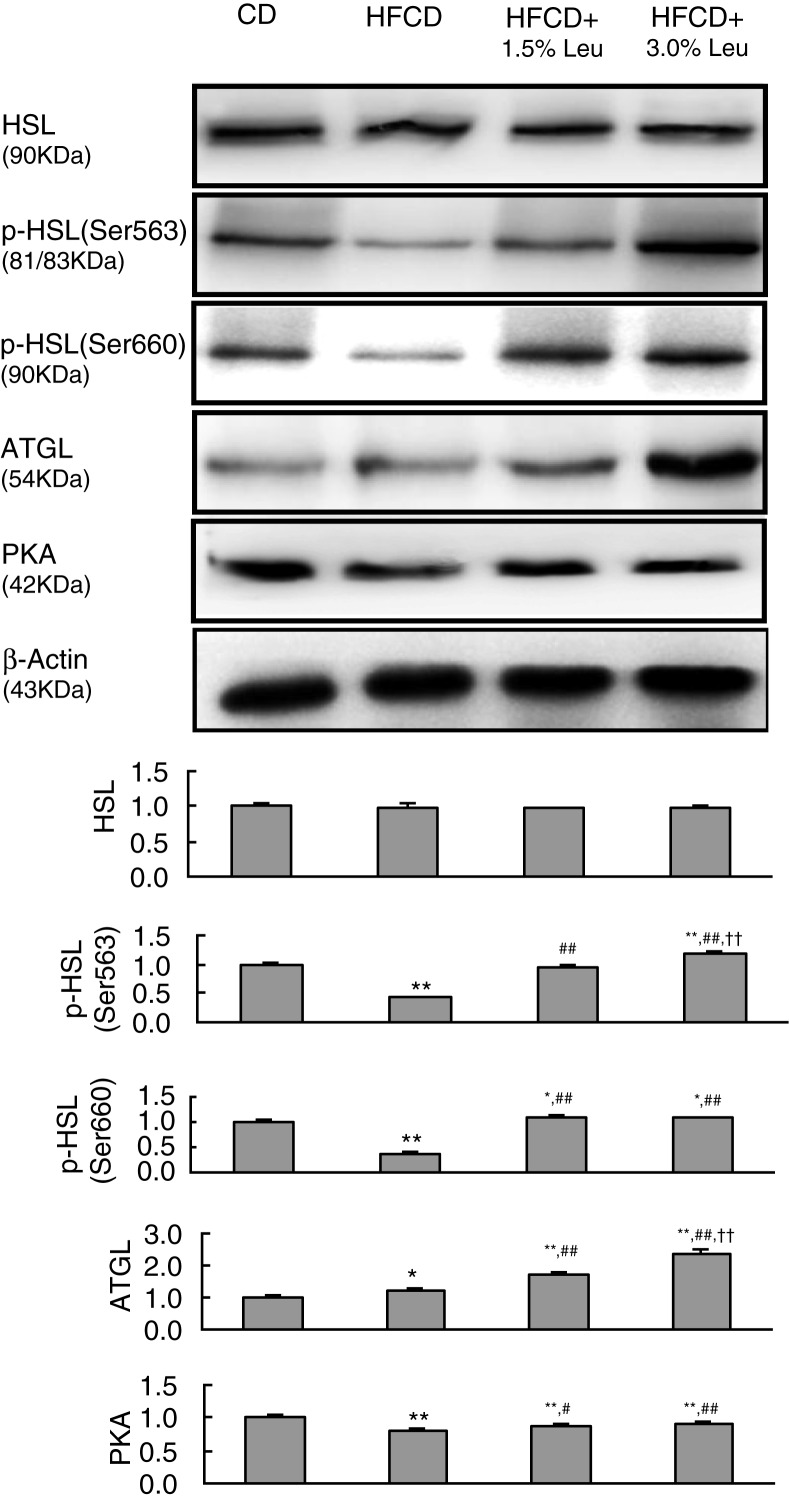
Chronic leucine supplementation improves lipid metabolism by increased adipocyte lipolysis in WAT of mice fed with HFCD. Values are means for three rats with SD represented by vertical bars. *p<0.05 versus CD group, **p<0.01 versus CD group, ^#^ *p*<0.05 versus HFCD group, ^##^ *p*<0.01 versus HFCD group, ^††^ *p*<0.01 versus HFCD+1.5% Leu group. WAT, white adipose tissue; CD, chow diet; HFCD, high-fat/cholesterol diet; Leu, leucine; HSL, hormone-sensitive lipase; Ser, serine; ATGL, adipose triglyceride lipase; PKA, cAMP-dependent protein kinase.

HSL and ATGL are two major lipases in adipocyte lipolysis. Their protein expression levels were measured in the epididymal WAT. Although total HSL expression had no effect, phosphorylated HSL was significantly decreased by HFCD. The protein expression of phosphorylated HSL and ATGL was markedly increased by leucine supplementation, even exceeding the CD group in the HFC+3.0% Leu group. Compared with the HFCD group, the protein expression of p-HSL(563), p-HSL(660), and ATGL increased by 2.80, 2.99, and 1.87 times in the HFC-3.0% Leu group. The abovementioned lipolysis mainly involves the PKA pathway. We also analyzed the expression of PKA in WAT and found that the decreased expression by HFCD was restored by additional leucine supplementation (Fig. 5).

**Fig. 6:**
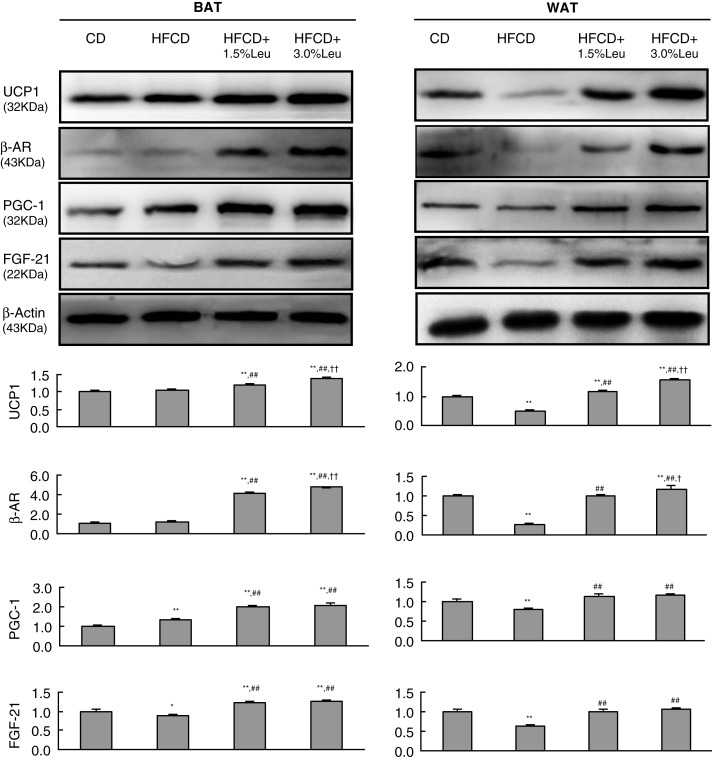
Chronic leucine supplementation improves lipid metabolism through thermogenic program and WAT browning in mice fed with HFCD. Values are means for three rats with SD represented by vertical bars. *p<0.05 versus CD group, **p<0.01 versus CD group, ^##^ *p*<0.01 versus HFCD group, ^†^ *p*<0.05 versus HFCD+1.5% Leu group, ^††^ *p*<0.01 versus HFCD+1.5% Leu group. BAT, brown adipose tissue; WAT, white adipose tissue; CD, chow diet; HFCD, high-fat/cholesterol diet; Leu, leucine; UCP1, uncoupling protein 1; β3AR, β3 adrenergic receptors; PGC-1, peroxisome proliferator-activated receptor g coactivator-1; FGF-21, fibroblast growth factor 21.

UCP1, β3AR, PGC-1, and FGF-21 were involved in the thermogenic program and WAT browning. In BAT, HFCD had no effect or even slightly upregulated their expression. Leucine supplementation remarkably increased the expression of the four proteins in a dose-response manner. Compared with the HFCD group, the protein expression levels of UCP1, β3AR, PGC-1, and FGF-21 in HFCD+3.0% Leu group increased by 1.34, 3.94, 1.54, and 1.45 times, respectively. Unlike BAT, HFCD caused the decreased expression of these proteins in WAT. However, leucine supplementation still increased their expression in a dose-response manner. Compared with the HFCD group, the expression of these proteins in the HFCD+3.0% Leu group increased by 3.06, 4.70, 1.20, and 1.69 times (Fig. 6).

## Discussion

In modern times, consuming high cholesterol in a HFD is increasingly popular worldwide and is strongly associated with conditions of obesity and other lifestyle diseases (22). This study demonstrated that dietary leucine prevents obesity induced by a HFD supplemented with additional cholesterol. The reduced obesity can be explained by improving lipid metabolism in the liver and adipose tissues.

In this study, leucine supplementation retarded weight gain without reducing energy intake, thereby suggesting a leucine-mediated increase in energy expenditure. Our observation was consistent with Zhang's and Binder's studies, in which leucine was consumed in drinking water along with a HFD (11, 17). Besides the decrease of fat and liver weights, histological examination also showed that HFCD-induced lipid accumulation in WAT and liver was markedly reduced by leucine supplementation. In fact, HFD feeding in rats for 3 weeks successfully reproduced the key features of non-alcoholic steatohepatitis, (23) and leucine supplementation improved this hepatic steatosis (13). Even in middle-aged adults during 14 days of bed rest, leucine prevented an increase in body fat percentage (24). Obesity results in dyslipidemia and dysglycemia. The beneficial effect of leucine supplementation on the lipid profile and circulating glucose/insulin levels has been extensively investigated in several obese animal models induced by a HFD (11–18). To better mimic hypercholesterolemia, additional cholesterol was contained in HFD to make HFCD in this study. As a result, leucine supplementation decreased the serum and hepatic TC and HOMA index. Therefore, with the reduced body weight and lipid accumulation, leucine supplementation generally improved lipid and glucose metabolism in this study. However, not all studies showed consistent results between body weight and glucose/lipid metabolism. For example, Macotela found that leucine supplementation causes a marked improvement in glucose tolerance and insulin signaling without altering weight gain and serum cholesterol in HFD-induced obese mice (13). Li even observed that leucine supplementation improves the insulin signaling pathway, while increasing the body weight and hepatic lipid accumulation in HFD-induced obese rats (16).

WAT is no longer considered a tissue mainly devoted to energy storage but is emerging as an active participant in regulating physiologic and pathologic processes through the synthesis and secretion of various adipokines. Excessive intake of dietary fat promotes adipocyte hypertrophy, altering their normal endocrine function (25). In this study, the inflammatory pathologic condition was found in HFCD-induced obese mice, in which increased levels of serum leptin, TNF-α, and IL-6 (proinflammatory adipokines) and reduced levels of adiponectin (main anti-inflammatory factor) were observed. Leucine supplementation decreased leptin, TNF-α, and IL-6, suggesting that inflammatory stress was attenuated. TNF-α is central to the chronic inflammatory state present in obesity (25). Reduced plasma TNF-α levels were observed in 20 overweight and obese subjects that received leucine-containing nutraceutical for 4 weeks (26). The mRNA level of TNF-α was also significantly reduced in perigonadal and subcutaneous adipose tissues of HFD-induced obese mice simultaneously supplemented with leucine for 8 weeks (13). Consistent with our study, Zhang also found that leucine intake decreases plasma leptin levels in obese mice (11). Our previous study further confirmed that chronic leucine supplementation improves HFD-induced leptin resistance by decreasing the circulating leptin level (19). Besides inflammation, oxidative stress is inextricably linked and plays major roles in the onset and development of obesity (27). In our previous study, leucine supplementation for 8 weeks resulted in a significant increase in the levels of total antioxidant capacity, superoxide dismutase (SOD), and glutathione, as well as a decrease in the malondialdehyde (MDA) level in insulin-resistant rats (28). Other studies also found that leucine supplementation inhibits the HFD-induced expression of manganese SOD in the liver and decreases the MDA level in the blood of animals fed with a HFD, which indicates that a chronic leucine supplementation may prevent HFD-induced oxidative stress (16). Together, the overall beneficial effect of leucine supplementation on lipid/glucose metabolism and adipokine inflammatory stress promoted us to investigate liver lipogenesis and adipose tissue lipolysis at the protein level.

HFD is known to upregulate LXRa, which then upregulates SREBP-1 to increase the activity of lipogenic enzymes, such as FAS and ACC, and accumulate TG in hepatocytes (29). Except through SREBP-1, LXRα directly regulates FAS and ACC (30). For the first time, we found that leucine reduced the expression of these two proteins in the liver, which was supported by the downregulation of their upstream proteins (LXRα and SREBP-1). Some studies observed FAS and ACC at the mRNA level in different tissues. In the study by Macotela, the expression levels of FAS and ACC mRNA increased in livers of HFD mice, and they were reverted to control levels when additional leucine was added (13). However, in already obese mice, leucine supplementation increased the FAS mRNA level, but not the FAS protein level in WAT (17). Interestingly, the downregulation of lipogenic enzyme at the mRNA level by leucine or other two BCAAs (isoleucine and valine) was also observed in the human intestinal cell line, NCI-H716 (31).

Catalytically active PKA phosphorylates downstream targets-HSL, which contribute to the initiation of ATGL, ultimately leading to the breakdown of TG (32). The effect of leucine on lipase-related protein has not been investigated. However, leucine is a potent activator of mTORC1 signaling (11, 16), which promotes lipolysis by acting on common lipolytic targets (e.g. HSL) through a pathway parallel to PKA (33). Whey protein is rich in leucine. Freudenberg found that high amounts of whey protein lead to increased lipolysis in white fat as indicated by increased gene expression of ATGL and HSL in mice fed with semisynthetic HFD for 20 weeks (34). In this study, leucine supplementation upregulated the expression of PKA, following the increase in HSL phosphorylation on Ser563, which is thought to promote the intrinsic catalytic activity of HSL, and HSL phosphorylation on Ser660, which is thought to promote the translocation of cytosolic HSL to the surface of the lipid droplet (32).

In rodents, BAT is the major organ responsible for adaptive thermogenesis during cold exposure (20). The UCP1 gene is known to be specifically expressed in BAT and is regarded as a BAT marker (35). The expression of UCP1 is driven by several transcriptional components, including the coactivator of PGC-1, which is strongly induced by β-adrenegic signaling (36). In addition, adipose-derived FGF21 acts to increase UCP1 expression by enhancing the PGC-1 protein level of adipose tissues (37). In this study, the expression levels of these proteins were detected in BAT and WAT. Interestingly, HFCD did not affect their expression in BAT, but remarkably decreased their expression in WAT. This finding suggested that high fat in diet had more profound effects on fat deposit in WAT. Recently, WAT browning in response to appropriate stimulation has become the target of numerous obesity therapeutics (20). An important characteristic of WAT browning is the overexpression of UCP1 in WAT. In fact, earlier studies showed that enhanced expression of UCP1 in WAT of mice can reduce obesity (38). Meanwhile, FGF21 is considered a novel adipokine that has recently been demonstrated to stimulate the browning of WAT in obese mice, even in healthy human (39, 40). Wang further found that the injection of recombinant murine FGF21 reduces IL-6, TNF-α, and leptin mRNA levels in WAT of monosodium glutamate-induced obese rats, with the improvement of glucose tolerance, lipid metabolic spectrum, and hepatic steatosis (41).

Until now, the effect of leucine on the expression of these proteins in adipose tissue has not been investigated. In Binder's previous study on already obese mice, leucine-supplemented mice exhibited a significant increase in mRNA expression of UCP1 in WAT, but not in BAT and muscle tissues (17). Vaughan found that leucine treatment induces a significant dose-dependent expression of PGC-1 in human rhabdomyosarcoma cells and mouse myoblast (C2C12) cells (42). This study demonstrated that leucine supplementation upregulated the expression levels of UCP1, β3AR, PGC-1, and FGF-21 in WAT, in addition to their overexpression in BAT in a dose-dependent manner. Thus, leucine supplementation both enhanced energy transformation from nutrients to heat directly in BAT and promoted WAT browning in WAT, thereby reducing obesity.

In conclusion, the present findings strengthen current evidence suggesting a beneficial role for leucine in the prevention of diet-induced obesity. Unlike previous studies, additional cholesterol in HFD and the beneficial effects of leucine on the lipid profile, glucose metabolism, and adipokine inflammatory stress were observed. This study focused on the systemic determination of protein expression related to lipid metabolism. This beneficial effect was ascribed to hepatic lipogenesis, adipocyte lipolysis, and WAT browning.
